# Temporal Variations of Water Chemistry in the Wet Season in a Typical Urban Karst Groundwater System in Southwest China

**DOI:** 10.3390/ijerph17072520

**Published:** 2020-04-07

**Authors:** Min Xiao, Zenglei Han, Sen Xu, Zhongliang Wang

**Affiliations:** 1 Tianjin Key Laboratory of Water Resources and environment, Tianjin Normal University, Tianjin 300387, China; 1810080004@stu.tjnu.edu.cn (Z.H.); wangzhongliang@vip.skleg.cn (Z.W.); 2 Institute of Surface-Earth System Science, Tianjin University, Tianjin 300072, China; xusen@tju.edu.cn

**Keywords:** water chemistry, dissolved inorganic carbon, carbon isotope, carbonate rock weathering, groundwater

## Abstract

It is important to investigate temporal variations of water chemistry for the purpose of improving water quality in karst groundwater systems. Groundwater samples were collected daily under various land uses of Guiyang. Major ions and stable carbon isotope composition of dissolved inorganic carbon (δ^13^C_DIC_) were analyzed to understand the biogeochemical processes. The water chemistry was dominated by Ca^2+^, Mg^2+^, HCO_3_^-^, and SO_4_^2-^, which mainly derived from the dissolution of carbonate rocks (limestone and dolomite) and oxidation of sulfide. The groundwater was defined as of the HCO_3_-Ca Mg and HCO_3_·SO_4_-Ca·Mg type, according to its hydrochemical characteristics. Results suggested that hydrochemical concentrations changed quickly, in response to rainfall events. The fast response revealed that karst groundwater was easily impacted by rainfall and anthropogenic inputs according to temporal variation of water chemistry. The distribution of DIC (dissolved inorganic carbon) and δ^13^C_DIC_ showed that DIC is mainly sourced from soil CO_2_(g) influx and carbonate dissolution. δ^13^C_DIC_ and major ions ratios suggested that carbonate minerals were dissolved by H_2_SO_4_ at groundwater in wooded area, contributing an important source for DIC due to the slight enrichment of heavy δ^13^C_DIC_. More negative δ^13^C_DIC_ values were observed after rainfall reflected the fact that soil CO_2_(g) and organic carbon oxidation influxes accounted for a large share during DIC formation. Various δ^13^C_DIC_ and hydrochemical patterns were observed under various land use and human activity conditions. Meanwhile, relative high nitrate loads were found in groundwater after rainfall, suggesting high anthropogenic inputs following rainwater as having side effects on water quality. This study suggests that water chemistry and isotopic proof provide a better understanding of water quality and carbon dynamics responding to rainfall events in the karst groundwater systems.

## 1. Introduction

Karst aquifers are important for supplying a large amount of fresh water for the whole world [1]. The karst landscape covers nearly 15% of the Earth’s land area, providing drinking water for 1/4 of the world’s population [2]. Groundwater is important for drinking and irrigation in the karst area, therefore, understanding the biogeochemical processes in karst areas is extremely significant [3,4,5,6]. Groundwater is an important part in the karstic ecosystem, but it is particularly sensitive to environmental changes and human activities [5,7,8]. With the acceleration of urbanization and industrialization, the eco-environment in karst areas is susceptible to being affected [9,10].

Stable carbon isotope (δ^13^C) and water chemistry parameters, can be acted as important tools to understand the biogeochemical processes in groundwater systems [5,6]. Karstic water is one of the most important groundwater resources supplying drinking water for more than 100 million people in Southwest China [8,11]. The aquifers provide a natural storage and movement for groundwater. In the studied region, the soil layers are thin and lack enough filtration for contamination. With the development of karst folding, a fault, and a fracture under the complex hydrogeological conditions, the contaminants on the earth’s surface could seep into aquifers directly or indirectly [9]. The special structure would result in changing the chemical composition drastically and leading to the deterioration of groundwater quality. Groundwater is a “hidden reservoir” of pollutants, and it is difficult to monitor. Springs are natural outcrops of groundwater and main drain [6,12]. The contaminants in aquifers will eventually show up in springs. Therefore, springs naturally become the monitoring medium for contaminants and the hydrogeochemical processes of groundwater in karst aquifers can be fully understood.

Hydrochemical composition is the basis for probing sources, runoff, and the eco-environment, and is important content for assessing groundwater resources [13]. It is of much guiding significance for utilizing and managing groundwater resources in the drainage basin, and the related protection of the eco-environment and construction [14,15]. The δ^13^C values of different carbon sources are differentiated obviously, and the environmental changes of groundwater would influence biogeochemical cycles of carbon and isotopic composition [4,5,6]. The average δ^13^C being added to the SO_4_^2−^-rich groundwater is −9.4‰, while the average δ^13^C being added is −11.6‰ for the HCO_3_-rich groundwater, due to dedolomitization and soil CO_2_(g) dissolution in groundwater [16]. The δ^13^C not only discern the sources of DIC in groundwater, but also identify the sources of contamination in groundwater as well as the biogeochemical processes, which influence the migration and fate of contaminants [5,10]. It was pointed out that the heaviest δ^13^C values of -8.5‰ in an aquifer containing gypsum, lowest δ^13^C values of −14.4‰ in an aquifer contaminated by organic pollutants [5]. The hydrochemical and isotopic detection of spring provides the method to obtain the information on recharge of groundwater, formation lithology, water-rock interaction, and land-use type that greatly impact the quality of ground water [5,6,11].

In order to clarify the regional recharge source for groundwater and hydraulic connections in a karst aquifer, we provide reasonable and effective suggestions for management and utilization of groundwater resources. The main objectives of this study are: (1) to analyze the hydrochemical characteristics responding to rainfall in this area, determine the subsequent reactions on the carbonate evolution, and further probe the hydrogeochemical evolutionary process in groundwater, thereby comprehensively understanding the impact of human activities on karst aquifer; (2) to understand the DIC sources in fresh groundwater and document the spatial and temporal characteristics for chemical components and stable carbon isotopes in Guiyang, Southwest China.

## 2. Materials and Methods

### 2.1. Study Area

The city of Guiyang is situated at 106°07´E-107°17´E longitude and 26°11´N-26°55´N latitude, in the centre of Guizhou Province, Southwest China. It covers an area of 2960 km^2^, with an average elevation of around 1400 m, characteristic of plateau hilly landforms [5,10]. Guiyang is located close to the boundary, separating the Changjiang River and Pearl River basins, occupying a wide karstic valley basin in the central part of Guizhou Province. Permian and Triassic carbonate rocks are widely distributed in the region; this kind of geology took an area over 70%, the lithology is mainly dolomite and limestone, and part of it contains organic- and sulfide-rich limestone [11]. Groundwater is rich in the terrain with karst development. The Guiyang basin is restricted and controlled by the combination of the topographic features of folding, a fault, and fracture structure planes, forming a karst underground water network model and groundwater accumulation area. Woodland, arable land, and urban land are the main land-use patterns within the sampling region. The climate in Guiyang city is subtropical with an average temperature of 16 °C and annual precipitation of about 1200 mm. A monsoonal climate often results in high precipitation during summer and much less during winter at the studied region. Rainfall is concentrated from May to August and the dominant recharge to ground water is dependent on rain water [8,9].

### 2.2. Sampling and Analytical Method

**Figure 1:**
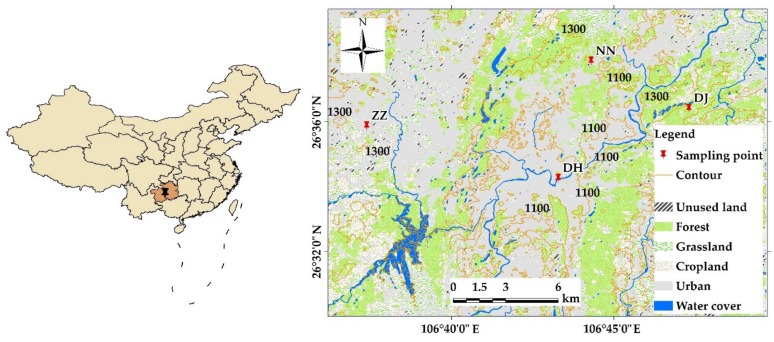
Sampling sites of groundwater and land utilization of Guiyang City, SW China.

Ground water samples were collected in August, 2011, corresponding to the high-flow seasons. Four sampling sites were selected situated at ZZ (Zhen Zhu, name of site) where it is newly urbanized with some cropland. NN (Nai Niu, name of site) is partially forested with some residents in the immediate surroundings, who cultivate crops and raise cows. ZZ and NN are gravity springs, with a mean discharge of 41.2 L/s and 1 L/s [11], respectively. DJ (Dong Jiao, name of site) is artesian spring having a discharge of 1571 L/s and located in a large area of woodland at the mountain zone [11]. DH (Di Hua, name of site) is a well with a discharge of 6.3 L/s and sits in the centre of the city (Figure 1). The Nanming River is the trunk stream, and the water flow direction is from southwest to northeast (Figure 1). Groundwater samples were collected every day in one month, and in total, over one hundred samples were collected. Meanwhile, the daily data of rainfall were collected synchronously during the sampling period through website of local meteorological bureau (http://gz.cma.gov.cn/). Temperature, electrical conductivity, and pH were measured in situ. Alkalinity was measured by titration with 0.02M HCl within 12 h. Major cations (Mg^2+^, Ca^2+^, Na^+^, and K^+^) were analyzed by atomic absorption spectrometry once samples were filtered and acidified, and anions (SO_4_^2−^, NO_3_^−^, and Cl^−^) were analyzed by high-performance liquid chromatography.

For the determination of δ^13^C_DIC_, samples were filtered through 0.45 μm cellulose acetate filter paper in order to avoid the potential influence of carbonate mineral particles. Some water samples were selected for isotopic analysis due to limitations of cost and time in this study. Using the method of Li et al. (2005) [5], 10 mL water sample was injected into glass bottles that were prefilled with 85% phosphoric acid and a magnetic stir bar by syringe. Then CO_2_ was extracted and purified into a vacuum line. Finally, the purified CO_2_ was transferred cryogenically into a tube welded for isotopic measurements. Carbon isotope ratios were determined on a Finnigan MAT 252 mass spectrometer (Thermo Finnigan GmbH Inc., Bremen, Germany) and reported in the δ notation relative to Vienna Pee Dee Belemnite (PDB) in per mill, (see Equation (1)):

*δ_sample_(‰)* = *((R_sample_* ÷ *R_VPDB_)−1)* × *1000* (1)

where *R* is the ^13^C/^12^C atomic ratio. Routine δ^13^C_DIC_ has an overall precision of 0.1‰. 10% of duplicate samples were measured, and the results showed that the differences were lower than the measurement accuracy.

## 3. Results

### 3.1. Hydrochemical Characteristics of Groundwater

The pH values of groundwater ranged from 6.0–8.0, which reflected the influence of the soil water with high CO_2_ concentrations. Under the observed pH conditions, bicarbonate (HCO_3_^−^) was the dominant DIC species [9,17,18] and the ion abundance was related to the dissolution equilibrium of the carbonate minerals. The average TDS (Total dissolved solids) values were 564 mg·L^−1^ for groundwater in summer [11]. The daily rainfall ranged from 0–89.6 mm, and averaged at 11.99 mm.

**Figure 2:**
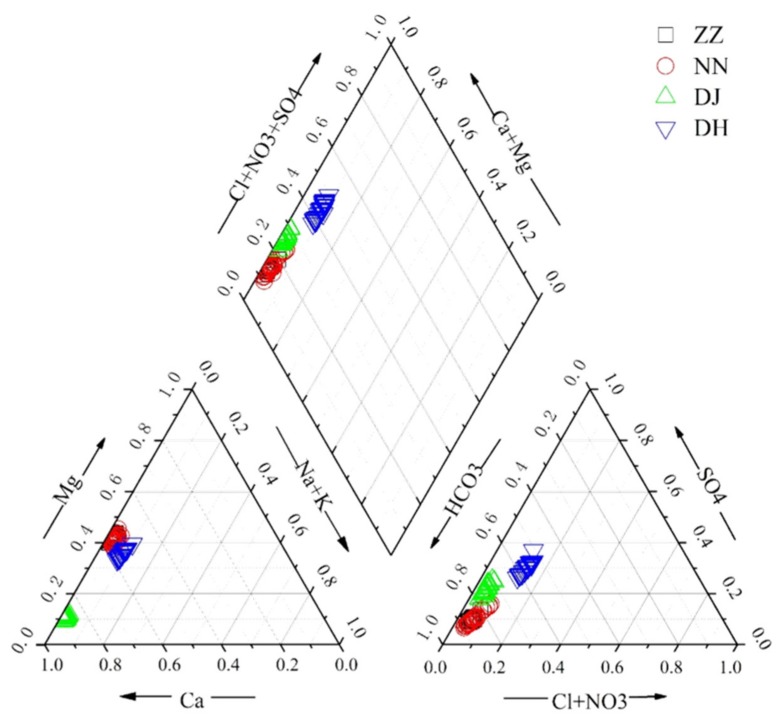
Trilinear diagram of hydrochemical composition in ground water samples in study area.

The major ion concentrations of samples are listed in Table S1. The charge balance errors between cations and anions in groundwater were equivalent to or less than 5%. It was obvious that Ca^2+^ and Mg^2+^ were the dominant cations, and HCO_3_^−^ and SO_4_^2−^ were the dominant anions (Figure 2), which accounted for 96% and 92.5% of cation and anion in milliequivalents (mEq), respectively. As illustrated in Figure 2, hydrochemical types of spring water in Guiyang were predominantly HCO_3_–Ca·Mg and HCO_3_·SO_4_–Ca·Mg. The chemical compositions of groundwater were characterized by a anion sequence of HCO_3_^−^ > SO_4_^2−^ > NO_3_^−^ + Cl^−^, accounting for 74%, 18%, and 7% of the total anions, respectively. The NO_3_^-^ level of groundwater at the residential district was higher than that of woodland. NO_3_^−^ was the most concentrated at DH with the proportion of 5% in total anions, with the absolute average amounts of 32.41 mg·L^−1^. Similar to NO_3_^−^, SO_4_^2−^ was the highest at DH, with a range from 114.4–188.9 mg·L^−1^ (averaging 144.7 ± 16.6 mg·L^−1^). The SO_4_^2−^ concentrations averaged at 25.9 ± 2.4, 33.35 ± 0.27, and 31.51 ± 4.1 mg·L^−1^ at ZZ, NN, and DJ, with ranges of 20.45–35.42, 20.73–50.13, and 23.86–38.01 mg·L^−1^, respectively.

HCO_3_^−^ was relative high at NN and DH, with averages of 66.52 ± 1.97 and 65.80 ± 0.92 mg C·L^−1^, whereas 55.69 ± 0.73 and 26.96 ± 1.01 mg C·L^−1^ were found at ZZ and DJ, respectively. Cl^-^ occupied the highest and lowest levels at DH and DJ, averaging 29.02 ± 2.47 and 1.60 ± 0.35 mg·L^−1^, respectively. Cl^−^ at ZZ and NN fell into the ranges of 3.5–5.8 and 3.47–5.64 mg·L^−1^, averaging 4.24 ± 0.46 and 4.42 ± 0.58 mg·L^−1^. For cations, the average concentrations of Ca^2+^ (64%) and Mg^2+^ (32%) were much higher than those of K^+^ and Na^+^ (4%). As the most abundant cation species, Ca^2+^ and Mg^2+^ at DH averaged 108.28 ± 10.06 and 41.95 ± 1.66 mg·L^−1^, respectively. Followed by NN, Ca^2+^, and Mg^2+^ have mean value of 70.58 ± 5.19 and 31.43 ± 0.68 mg·L^−1^, respectively. Ca^2+^ and Mg^2+^ have a range of 56.83–66.33 mg·L^−1^ and 25.88–29.28 mg·L^−1^ at ZZ, with average values of 61.57 ± 2.34 mg·L^−1^ and 27.66 ± 0.63 mg·L^−1^, respectively. Levels of 47.18–56.35 mg·L^−1^ for Ca^2+^ and 3.38–4.38 mg·L^−1^ for Mg^2+^ were observed, with averages of 52.86 ± 2.34 and 3.83 ± 0.29 mg·L^−1^ at DJ, respectively. Na^+^ and K^+^ were the lower cationic species in the total cations and the gross of Na^+^ + K^+^ ranged from 18.86–22.43 mg·L^−1^, 3.61–9.99 mg·L^−1^, 2.71–5.85 mg·L^−1^, and 1.4–2.13 mg·L^−1^ at DH, NN, ZZ, and DJ, with mean values of 20.84 ± 1.03, 5.4 ± 1.68, 3.02 ± 0.58, and 1.66 ± 0.17 mg·L^−1^, respectively.

### 3.2. DIC and δ^13^C_DIC_

DIC concentrations were relative high at NN and DH, ranging from 60.91–68.4 and 62.9–66.96 mg C·L^−1^, with averages of 66.52 ± 1.97 and 65.8 ± 0.92 mg C·L^−1^, respectively. Narrow ranges of 52.3–56.54 and 25.31–28.84 mg C·L^−1^ with averages of 55.69 ± 0.73 and 26.96 ± 1.01 mg C·L^−1^ were observed at ZZ and DJ. The δ^13^C_DIC_ averaged −9.84‰ ± 0.05‰, −10.83‰ ± 0.19‰, −10.35‰ ± 0.13‰, and −11.30‰ ± 0.21‰ at ZZ, NN, DJ, and DH, and they correspondingly ranged from −9.97‰ to −9.73‰, −11.05‰ to −10.45‰, −10.49‰ to −10.17‰, and −11.56‰ to −10.93‰, respectively.

## 4. Discussion

### 4.1. Characteristics of Groundwater Chemistry and Water–Rock Reactions

**Figure 3:**
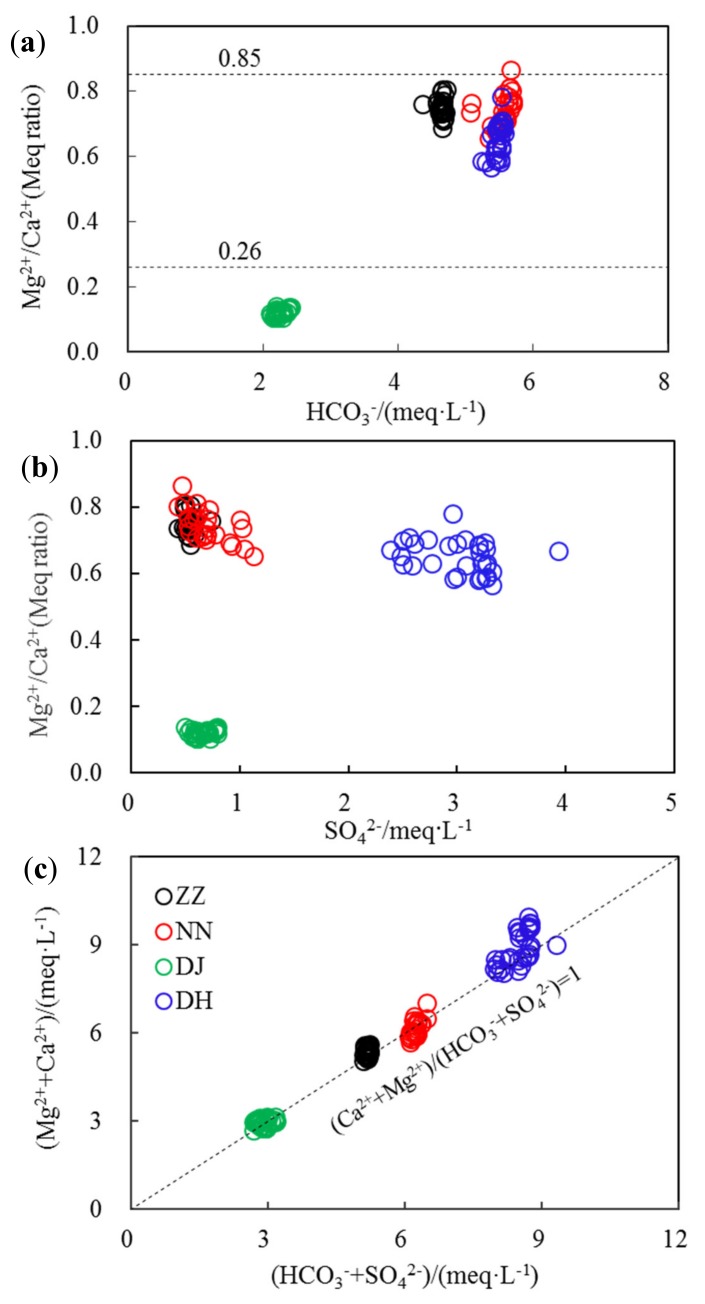
Explanation for regular pattern of hydrochemical composition in groundwater of Guiyang (**a**)—denotes the ionic source of different lithology as calcite or dolomite; (**b**)—denotes the gypsum contribution to ionic composition; (**c**)—denotes the balance of main cations and main anions; meq·L^−1^ denotes the unit of ionic level by milliequivalent per liter; Meq Ratio denotes the ratios of ionic contents by milliequivalent units.

The molar ratio of Mg^2+^/Ca^2+^ is a good indictor to reflect the lithology of aquifers that groundwater flows through [4,9]. When this value is much higher than 0.85, it indicates the dominant presence of dolomite sources. If this ratio was between 0.01 and 0.26, hydrochemical components in groundwater were mainly derived from limestone sources [9]. In this study, the averaged Mg^2+^/Ca^2+^ ratio was 0.71, much higher than 0.26 but lower than 0.85 for ZZ, NN, and DH, indicating that the hydrochemical components were mainly from the dissolution of both dolomite and limestone (Figure 3a). This ratio was much lower than 0.26 at DJ, with an average of 0.12, suggesting a limestone source at this sampling site. In addition, the molar ratios of Mg^2+^/Ca^2+^ at ZZ, NN, and DH varied in a narrow range, from 0.6–0.8. Whereas SO_4_^2−^ varied in a large range, the molar ratio at DJ was much lower (Figure 3b). The result indicated that gypsum should not be considered as the main source of SO_4_^2−^ and Ca^2+^. SO_4_^2−^ in groundwater can be sourced from oxidation of sulfide minerals, which are widely distributed in coal-containing strata [11]. In addition, acid rain events often occur in Guiyang, characterized by a high H_2_SO_4_ content, and this is probably also an important source of SO_4_^2−^ [19].

The Ca^2+^ and Mg^2+^ concentrations were much higher at DH than those found in other sites, because of the employment of exogenous acid increasing carbonate dissolution in summer. Moreover, domestic sewage discharge, sulfuric acid rain, etc., resulted in increased H^+^ and accelerated carbonate weathering [20,21]. As elucidated in Figure 3c, (Mg^2+^ + Ca^2+^)/(HCO_3_^−^ + SO_4_^2−^) was basically around 1, implying that dissolution of carbonate and S-containing minerals co-affected the hydrochemical compositions of groundwater in Guiyang. It is in agreement with previous studies in Southwestern China [22,23]. This ratio occasionally (from 17–28 August) slightly exceeded 1 at DH, which means that this system needed other anions to balance the excess Ca^2+^ and Mg^2+^, especially since this ratio rose to 1.14 at 23rd and 24th of August. Another possible reason for balance would be high nitrate from nitrification [21] due to high human activities [8] in studied sites. Major cations and anions have relative low concentrations in this study when compared to data at these fours sits at the dry season [5], which suggested dilution processes play an important role in water chemistry at karst ground water.

**Figure 4:**
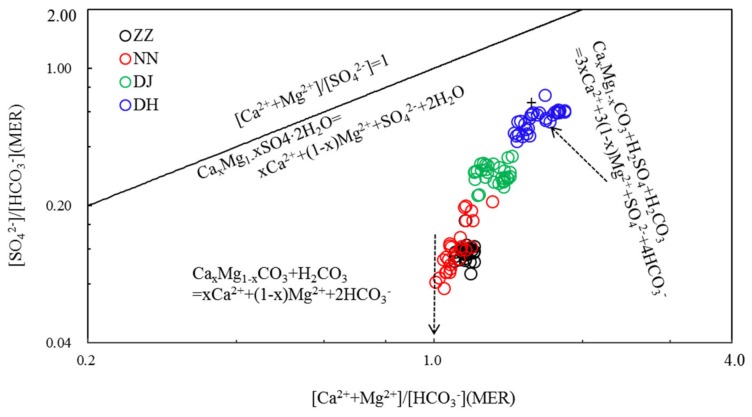
Variations of SO_4_^2−^/HCO_3_^−^ with (Ca^2+^ + Mg^2+^)/HCO_3_^-^ equivalent ratios in the groundwater of Guiyang (MER—milliequivalent ratio).

Both H_2_CO_3_ and H_2_SO_4_ can play a part in minerals’ dissolution according to reaction formula (Equation (2)) [22,24]. Figure 4 shows the variations of (Ca^2+^ + Mg^2+^)/HCO_3_^−^ vs. SO_4_^2−^/HCO_3_^-^ equivalent ratios for the water samples. The transformation mechanism for them was interpreted as follows:

Ca_x_Mg_1−x_CO_3_ + H_2_SO_4_ + H_2_CO_3_ = 3xCa^2+^ + 3(1 − x)Mg^2+^ + SO_4_^2-^ + 4HCO_3_^-^ (2)

The chemical reactions would be most likely responsible for the water chemistry. For water samples with unified (Ca^2+^ + Mg^2+^)/HCO_3_^−^ and low SO_4_^2−^/HCO_3_^−^ ratios, carbonate dissolution by H_2_CO_3_ dominated the mineral/water interaction. With the increasing SO_4_^2−^/HCO_3_^−^ ratios, the (Ca^2+^ + Mg^2+^)/HCO_3_^−^ ratio also increased, and more SO_4_^2−^ was needed to balance the excess Ca^2+^ and Mg^2+^ in the water samples.

When carbonate dissolution by both H_2_CO_3_ and H_2_SO_4_, water should have a SO_4_^2−^/HCO_3_^−^ ratio of 0.5 and a (Ca^2+^ + Mg^2+^)/HCO_3_^−^ ratio of 1.5, as shown by Figure 4. When both SO_4_^2−^/HCO_3_^−^ and (Ca^2+^ + Mg^2+^)/HCO_3_^−^ ratios continued to increase, dissolution of gypsum was needed to balance the negative and positive ions. The covariation of (Ca^2+^ + Mg^2+^)/HCO_3_^−^ and SO_4_^2−^/HCO_3_^−^ ratios can also be interpreted as binary mixing of HCO_3_^−^ type and SO_4_^2−^ type water, revealing that water chemistry originated mainly from carbonate dissolution by H_2_CO_3_ and H_2_SO_4_.

**Figure 5:**
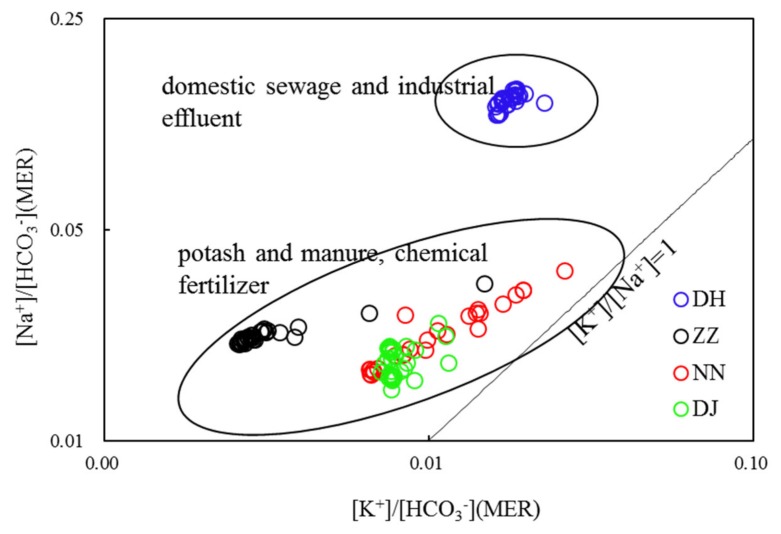
Correlations of HCO_3_^−^ normalized by the Na^+^ and K^+^ values (molar ratios) of groundwaters in Guiyang (MER—milliequivalent ratio).

The proportion of elements can be used to investigate the water–rock interaction and the chemical evolution of groundwater [9]. Generally, studying on the affinity of water chemistry throws light on the geochemical characteristics of the aquifer. The incorporation of industrial and urban NaCl and potash fertilizers, manure and N-containing fertilizers used in agricultural production into aquifers will lead to the increase of K^+^ and Na^+^ in groundwater. As shown in Figure 5, the sewage samples at DH showed low K^+^/Na^+^ ratios, meaning that sewage was strongly enriched in Na^+^. The relationship between Na^+^/HCO_3_^-^ and K^+^/HCO_3_^-^ at ZZ and NN indicated that groundwater has received significant agricultural input. The correlation between Na^+^/HCO_3_^−^ and K^+^/HCO_3_^−^ was not observed at DJ and DH, and the characteristic of the hydrochemical components will be explained below.

### 4.2. Response of Groundwater to Rainfall Events and Evolution of Water Quality

**Figure 6:**
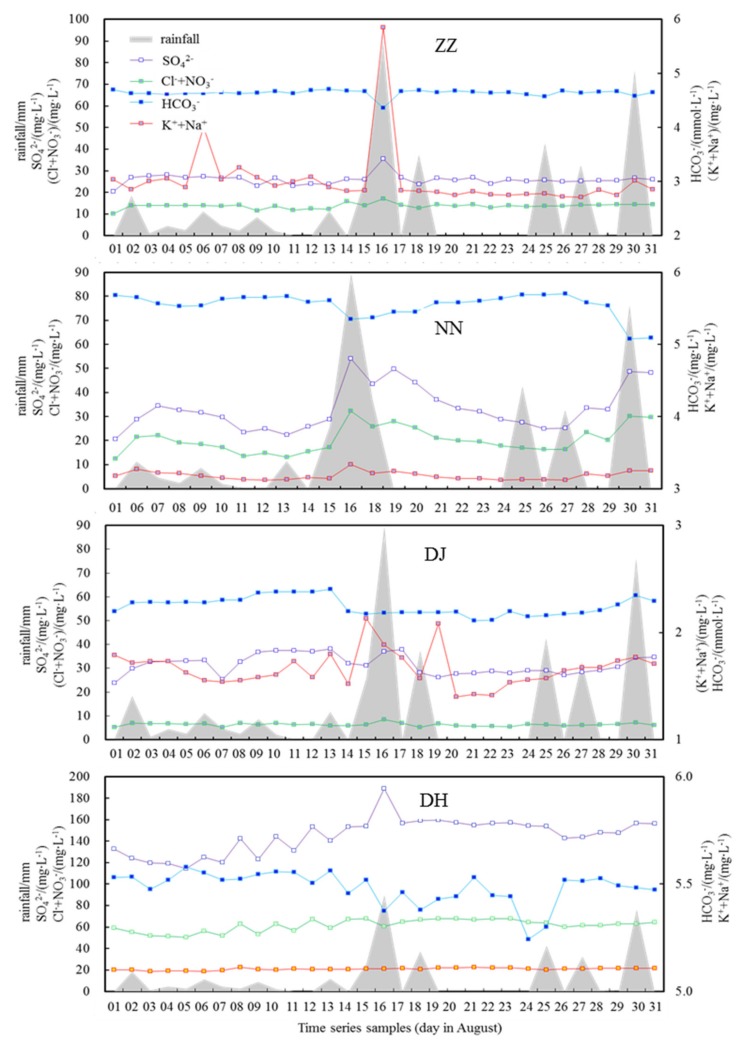
The temporal variations of major ions of different groundwater sampling sites and rainfall along sampling days at Guiyang.

Cl^−^ is conservative, showing an increasing trend from the upper reaches of the Nanming River due to anthropogenic inputs [11]. Halite and marine sources were not the main sources of Cl^−^, but from agricultural fertilizers, domestic sewage, animal manure, and Cl_2_ disinfection treatment of tap water [25]. Figure 6 shows the temporal variations of major ions during the sampling period. The ions, which were the result of anthropogenic inputs, included NO_3_^−^, Cl^−^, Na^+^, K^+^, and performed consistently with that of SO_4_^2−^ at ZZ, NN, and DH (R^2^ = 0.75, *p* ≦ 0.01), with an exception at DJ, which was covered with a large area of vegetation (Figure 6). Once the concentration of SO_4_^2−^ exceeds 100 mg·L^−1^ (average level 144.8 mg·L^−1^), this means that the anthropogenic input of SO_4_^2−^ is from the sewage effluent rather than the natural sources at DH. SO_4_^2−^ at ZZ, NN, and DH exhibited obvious anticorrelations with HCO_3_^−^ (R^2^ = 0.72, *p* ≦ 0.01), which implied that H_2_SO_4_ played an important role in DIC production, mediating in mineral weathering such as the oxidation of S-containing coal strata or organic matter in sewage effluent [26]. Contrarily, SO_4_^2−^ at DJ varied correspondingly to HCO_3_^-^ (R^2^ = 0.43, *p* ≦ 0.01), due to the carbonate dissolution by H_2_SO_4_ and H_2_CO_3_ in the region with less anthropogenic input that led to SO_4_^2−^ and HCO_3_^−^ production of similar variation trends (Figure 6). This viewpoint further confirmed the finding that SO_4_^2−^ originated from the decomposition of manure, fertilizer, and municipal sewage in groundwater samples at ZZ, NN, and DH. DJ is situated in woodland and has not been influenced by industrial and residential effluent. However, it is an exception at DJ, in that HCO_3_^−^ and SO_4_^2−^ exhibited a consistent trend and without any correlations to other ions, demonstrating that SO_4_^2−^ had a significant natural source from mineral weathering according to reaction formula (Equation (2)).

Atmospheric precipitation is the main source of regional groundwater, and surface water is hydraulically closely linked to groundwater [25]. It has been reported that the average concentrations of major ions in summer were generally lower than other seasons in Guiyang groundwater, due to the influx of infiltrated rain with low solutes contents [11]. As illustrated in Figure 6, rainfall influenced the ionic compositions in that HCO_3_^−^ decreased abruptly with the heavy rain at 16th, August. Obviously, HCO_3_^−^ from natural sources was acutely diluted by the heavy rainfall due to the large rain exchange flux, and impacted by the removal of the total quantity from weathered rocks [5]. However, frequent rainfall did not change ion concentration largely in the following days due to adaption of water–rock reaction in high rainfall periods. Whereas SO_4_^2−^, K^+^, Na^+^, and NO_3_^−^ increased quickly under heavy rain, similar to the findings of other study [27] that this group of ions were from anthropogenic discharge, washed and infiltrated into river water and elevated significantly during rainfall events. Although SO_4_^2−^ at DJ originated similarly from mineral dissolution, which was not as susceptible to being affected by heavy rain as HCO_3_^−^, it is probably due to occasional manure and fertilizer decomposition, or else a contribution from acid rain.

The relationship between ions and meteoric water could be used to constrain the sources of contaminants and their conversions. The hydrogeological features of karst areas permit the rapid penetration of pollutants into groundwater, so karst ground waters are more vulnerable to being polluted from human activities than other types of aquifers [25]. Groundwater in karst areas has been increasingly polluted by penetration of pollutants, e.g., uncontrolled urban sewage effluent and intensive utilization of fertilizers on cultivated land [15,25]. For HCO_3_^−^, Ca^2+^, and Mg^2+^, there is a relative low ion content in rainwater compared with that in groundwater [19]. The infiltrated meteoric water diluted and eroded the concentrations of HCO_3_^−^, Ca^2+^, and Mg^2+^, but NO_3_^−^, SO_4_^2−^, K^+^, and Na^+^ were eluted out and leached into groundwater mainly due to anthropogenic input from agricultural fertilizer, industrial waste, and domestic sewage following the rainfall [21]. Thus, relative high flow during the rainfall periods can lead to rapid leaching of surficial fertilizers/manure into the groundwater system through conduits due to weak buffering capacity in the studied area. Of course, the different response to rainfall in various groundwater sites suggested different role of high heterogeneity of karst system and extent of human activities as well as multi biogeochemical processes.

### 4.3. DIC Sources and the Controlling Factors in Groundwater

In the groundwater, the sources of DIC are from (1) the dissolution and dissociation of CO_2_(g), mainly from root respiration and soil organic matter decay, which is infiltrated in subsurface water systems, and (2) the dissolution of carbonates by organic acids excreted by plant roots, and carbonic acid produced by the dissolution of the CO_2_ in water [13,28,29]. The dissolution of carbonates (calcite and dolomite) is a major source of Ca^2+^ and Mg^2+^, which also generate DIC in groundwater. These are expressed as the following reactions (Equation (3)) and (Equation (4)).

CaCO_3_ + H_2_O+CO_2_(g) ⇿ Ca^2+^ + HCO_3_^−^ (3)

CaMg(CO_3_)_2_ + 2H_2_O + 2CO_2_ ⇿ Ca^2+^ + Mg^2+^ + 4HCO_3_^−^ (4)

The DIC sources are also likely to be organic residuals that are degraded by microbes, which will generate DIC to the water [18,26]. A large of aerobic and anaerobic populations predominates the downgradient from the pit water contaminated source [26]. As CO_2_(g) is generated by the microbiological degradation of hydrocarbons, which added to the CO_2_(g) generated from the organic matter respiration in the root zone, then the soil CO_2_ dissolves carbonates to produce DIC in the aquifer. However, hydrocarbon microbial mineralization should not be an important source of DIC [16]. Guiyang suffered from acid rain, which contains high sulfate [19]. Since sulfide minerals oxidation in the coal-containing strata [9], sulfuric acid plays a significant role in dissolving the carbonate rocks in this study area, producing DIC in groundwater.

Soil CO_2_(g), from respiration of C-3 plant, dissolving in groundwater produces DIC, with a δ^13^C_DIC_ value of −23‰ [4,30]. It is reported that Triassic and Permian limestone and dolomite are the most widely distributed strata in the studied area, with δ^13^C values ranging from −1.8–4.8‰ [31]. Supposing that δ^13^C only originated from soil CO_2_ influx and carbonate rock weathering, the δ^13^C value should be −11‰ [4,23]. δ^13^C values were much higher than −11‰ at ZZ, NN, and DJ, whereas it fluctuated around −11‰ or even lower at DH. The δ^13^C values for all samples were lower than −9‰, suggesting the contribution of soil CO_2_ influx. DIC generally has more negative δ^13^C value comparing to the data in the dry season from previous study [5], indicating DIC in the wet season has a relatively enriched biogenic origin relative to the dry season.

**Figure 7:**
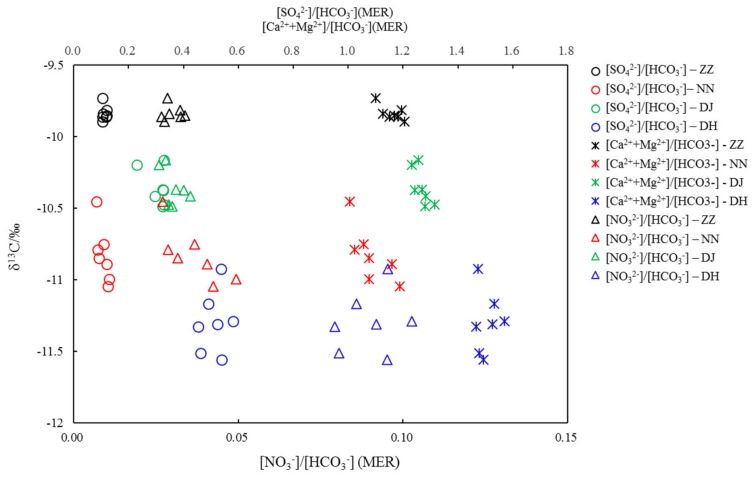
Covariations between δ^13^C_DIC_ and concentrations of SO_4_^2-^, (Ca^2+^+Mg^2+^), and NO_3_^-^ normalized by HCO_3_^-^ (MER: milliequivalent ratio).

In this study, HCO_3_^-^ is more than 95% of DIC based on water chemistry and pH. Milliequivalent ratios (MER) of (Ca^2+^ + Mg^2+^)/HCO_3_^−^ of the groundwater in Guiyang were in the range of 1–2, averaging 1.16, 1.11, 1.32, and 1.63 at ZZ, NN, DJ, and DH, respectively. The SO_4_^2−^/HCO_3_^−^ ratios averaged 0.12, 0.13, 0.29, and 0.55 at ZZ, NN, DJ, and DH, respectively. The relationship between (Ca^2+^ + Mg^2+^)/HCO_3_^−^ and SO_4_^2−^/HCO_3_^−^ indicated that H_2_SO_4_ acted a role in the mineral weathering. In addition, HCO_3_^-^ normalized SO_4_^2−^ value decreased in the rank of DH > DJ > NN > ZZ, suggesting that H_2_SO_4_ was decreasingly involved in carbonate weathering, as elucidated in Figure 4 [6]. Samples at DH showed a relatively high SO_4_^2−^/HCO_3_^−^ ratio and the lowest δ^13^C value (Figure 7). These results corresponded to the discussion of previous study [11], showing that since SO_4_^2−^ was mainly derived from oxidation of sulfide minerals or mineralization of organic sulfur. In this study, groundwater would be characterized by a high SO_4_^2−^/HCO_3_^−^ ratio, but δ^13^C-depleted DIC value. The carbonate evolution with the average δ^13^C_DIC_ of −11.3‰ at DH was undergoing in a closed system from carbonate weathering by soil CO_2_(g) [16]. The δ^13^C of −9.8‰ at ZZ was similar to the δ^13^C of −9.4‰ in SO_4_^2−^-rich water, both due to dedolomitization referred to previous study [16], that high δ^13^C values were relative to the result of the other sampling sites. [NO_3_^−^]/[HCO_3_^−^] averaged 0.03, 0.05, 0.03, and 0.1, likewise, displayed the same variation trend with (Ca^2+^+Mg^2+^)/HCO_3_^−^ at NN and DH, resulting in the lower δ^13^C values at NN and DH (Figure 7). It was inferred that carbonate weathering could be driven by nitric acid. However, NO_3_^-^ might play a minor role in shifting δ^13^C-depleted values due to oxidization of organic matter in sewage effluent at site of DH.

As shown in Figure 7, NO_3_^−^ played an important role in HCO_3_^−^ dynamics only at NN, reflecting on the anticorrelation between them (R^2^ = 0.72, *p* ≦ 0.01). It was postulated that the transformation from organic carbon to DIC occurred with NO_3_^−^-mediating in the redox reactions, shifting negative δ^13^C_DIC_ values. Probably this could be taken as a plausible explanation for the lower δ^13^C value at NN than that at DJ. Utilization of manure and nitrogenous fertilizer produces H^+^ during their oxidation, and sulfides in coal seams are oxidized into sulfuric acid, both of which promote the weathering of carbonate rocks and result in high NO_3_^−^ and SO_4_^2−^ concentrations and δ^13^C-enriched DIC values in karst waters [21,23,25]; therefore, nitric acid and sulfuric acid were also the driving factors for mineral dissolution and heavy δ^13^C. In addition, mineral dissolution and organic matter decomposition caused the δ^13^C values to fluctuate [17,32]; δ^13^C values deviated off −11‰ toward a more positive value, significantly negatively correlated to NO_3_^−^/HCO_3_^−^ and SO_4_^2−^/HCO_3_^−^ ratios at NN (R^2^ = 0.61, *p* ≦ 0.01), explained by the above elaboration that carbonate dissolution was significantly shifted by H_2_CO_3_, H_2_SO_4_, and HNO_3_, along with organic matter being oxidized into DIC by SO_4_^2−^ and NO_3_^−^. This phenomenon was not observed at ZZ, DJ, and DH, which further demonstrated that carbonate weathering by SO_4_^2−^ and NO_3_^−^ and organic carbon transformation were not the only factors influencing the δ^13^C_DIC_. The δ^13^C_DIC_ value of groundwater in Guiyang is mainly influenced by anthropogenic inputs following by carbonate rock weathering and soil CO_2_(g) influx. δ^13^C_DIC_ decreased with the increasing quotients of SO_4_^2−^ and NO_3_^−^ vs. HCO_3_^−^ at these sampling sites, indicating that H_2_SO_4_ and HNO_3_ were not only involved in carbonate mineral dissolution, but also in organic matter oxidation. In addition, soil CO_2_(g) influx shift DIC with δ^13^C-depleted values.

## 5. Conclusions

This study investigated temporal and spatial variations of water chemistry at the rainy season in a typical karst groundwater system, SW China. Groundwater had a high content of Ca^2+^, Mg^2+^, HCO_3_^−^, and SO_4_^2−^ in the study region, which accounted for more than 90% of total ions and were largely derived from dissolution of carbonate rocks. The chemical composition of ground water was mainly controlled by rock dissolution. Anthropogenic inputs into the groundwater mainly include SO_4_^2−^, Cl^−^, and NO_3_^−^, according to the chemical composition of municipal sewage in Guiyang. Domestic and industrial waste was the main contaminant sources of groundwater in urban areas, while agricultural fertilizers and the transformation products polluted groundwater in suburbs.

The study showed that carbonate evolution in freshwater aquifers could be traced by investigating the major ions and δ^13^C_DIC_. The coupled analysis of δ^13^C_DIC_ and hydrochemical parameters is an effective approach to explore the biogeochemical processes of carbon and trace the sources of groundwater pollutants in karst areas. Groundwater sampled from the city center (DH) showed the highest ionic level and lowest δ^13^C values relative to the other samples due to undergoing different carbonate evolution.

## Supplementary Materials

The following are available online at https://www.mdpi.com/1660-4601/17/7/2520/s1, Table S1: The major ions concentration in groundwater samples at Guiyang.
