# Supplementary material for: Temporal Variations of Water Chemistry in the Wet Season in a Typical Urban Karst Groundwater System in Southwest China
Source: Int J Environ Res Public Health. 2020 Apr 7;17(7):2520. doi: 10.3390/ijerph17072520 (PMC7177296; doi:10.3390/ijerph17072520)
Supplement: Supplementary file 1 [file ijerph-17-02520-s001.pdf]

Table S1. The major ions concentration in groundwater samples at Guiyang.

sample ID	Cl^-	NO_3^-	SO_4^{2-}	HCO_3^-	Ca^{2+}	K^+	Mg^{2+}	Na^+
				mmol/l				
ZZ01	0.10	0.11	0.21	4.69	1.60	0.01	1.18	0.11
ZZ02	0.13	0.15	0.28	4.63	1.57	0.01	1.15	0.10
ZZ03	0.13	0.15	0.29	4.63	1.62	0.01	1.17	0.11
ZZ04	0.13	0.15	0.29	4.61	1.54	0.02	1.15	0.11
ZZ05	0.13	0.15	0.28	4.63	1.58	0.01	1.15	0.10
ZZ06	0.14	0.15	0.29	4.63	1.58	0.03	1.17	0.12
ZZ07	0.13	0.15	0.28	4.65	1.60	0.01	1.18	0.11
ZZ08	0.12	0.16	0.28	4.63	1.54	0.02	1.16	0.11
ZZ09	0.11	0.12	0.24	4.64	1.52	0.01	1.22	0.11
ZZ10	0.12	0.15	0.28	4.67	1.52	0.01	1.17	0.10
ZZ11	0.11	0.13	0.24	4.63	1.61	0.01	1.19	0.11
ZZ12	0.11	0.14	0.25	4.69	1.47	0.01	1.16	0.11
ZZ13	0.11	0.13	0.25	4.71	1.44	0.01	1.16	0.10
ZZ14	0.16	0.16	0.27	4.68	1.65	0.01	1.17	0.10
ZZ15	0.12	0.15	0.27	4.67	1.56	0.01	1.16	0.10
ZZ16	0.13	0.20	0.37	4.36	1.43	0.06	1.09	0.15
ZZ17	0.12	0.16	0.28	4.67	1.53	0.01	1.13	0.10
ZZ18	0.11	0.14	0.25	4.68	1.56	0.01	1.14	0.10
ZZ19	0.12	0.16	0.28	4.65	1.49	0.01	1.15	0.10
ZZ20	0.12	0.15	0.27	4.68	1.57	0.01	1.15	0.10
ZZ21	0.12	0.16	0.28	4.66	1.52	0.01	1.15	0.10
ZZ22	0.11	0.14	0.25	4.64	1.55	0.01	1.15	0.10
ZZ23	0.12	0.15	0.27	4.65	1.66	0.01	1.14	0.10
ZZ24	0.12	0.15	0.26	4.61	1.54	0.01	1.13	0.10
ZZ25	0.12	0.15	0.27	4.57	1.50	0.01	1.14	0.10
ZZ26	0.10	0.16	0.26	4.68	1.51	0.01	1.08	0.10
ZZ27	0.11	0.17	0.26	4.64	1.54	0.01	1.17	0.10
ZZ28	0.11	0.17	0.27	4.66	1.52	0.01	1.16	0.10
ZZ29	0.11	0.17	0.27	4.67	1.53	0.01	1.15	0.10
ZZ30	0.11	0.17	0.28	4.58	1.47	0.02	1.13	0.10
ZZ31	0.11	0.17	0.27	4.65	1.42	0.01	1.14	0.10
NN01	0.12	0.13	0.22	5.68	1.65	0.05	1.32	0.15
NN06	0.14	0.27	0.30	5.65	1.63	0.10	1.32	0.17
NN07	0.15	0.27	0.36	5.56	1.67	0.08	1.32	0.15
NN08	0.13	0.23	0.34	5.53	1.93	0.08	1.36	0.15
NN09	0.13	0.22	0.33	5.53	1.88	0.06	1.34	0.13
NN10	0.12	0.21	0.31	5.63	1.67	0.05	1.31	0.11
NN11	0.10	0.16	0.24	5.65	1.59	0.04	1.29	0.10
NN12	0.11	0.18	0.26	5.65	1.75	0.04	1.30	0.09
NN13	0.10	0.15	0.23	5.66	1.53	0.04	1.32	0.10
NN14	0.11	0.19	0.27	5.59	1.74	0.05	1.27	0.11
NN15	0.12	0.21	0.30	5.61	1.84	0.05	1.31	0.11
NN16	0.16	0.43	0.56	5.35	2.12	0.14	1.39	0.20
NN18	0.14	0.34	0.45	5.37	1.90	0.08	1.32	0.14
NN19	0.15	0.37	0.52	5.45	1.94	0.09	1.31	0.16
NN20	0.14	0.33	0.46	5.45	1.88	0.07	1.28	0.14
NN21	0.13	0.27	0.38	5.58	1.83	0.06	1.32	0.12
NN22	0.13	0.25	0.35	5.58	1.84	0.05	1.33	0.11
NN23	0.13	0.24	0.33	5.60	1.85	0.04	1.33	0.11
NN24	0.12	0.22	0.30	5.64	1.70	0.04	1.31	0.10

NN25	0.11	0.21	0.29	5.69	1.75	0.04	1.33	0.10
NN26	0.10	0.21	0.26	5.68	1.70	0.04	1.31	0.10
NN27	0.10	0.21	0.26	5.70	1.70	0.04	1.30	0.10
NN28	0.13	0.30	0.35	5.58	1.70	0.08	1.30	0.13
NN29	0.12	0.26	0.34	5.54	1.73	0.06	1.27	0.13
NN30	0.13	0.41	0.51	5.08	1.69	0.10	1.25	0.16
NN31	0.13	0.40	0.50	5.09	1.66	0.10	1.27	0.16
DJ01	0.05	0.06	0.25	2.20	1.18	0.02	0.16	0.04
DJ02	0.05	0.08	0.31	2.28	1.22	0.02	0.16	0.05
DJ03	0.05	0.08	0.34	2.29	1.23	0.02	0.15	0.05
DJ04	0.05	0.08	0.34	2.28	1.23	0.02	0.15	0.05
DJ05	0.05	0.08	0.35	2.29	1.24	0.02	0.16	0.04
DJ06	0.05	0.08	0.35	2.28	1.27	0.02	0.15	0.04
DJ07	0.04	0.06	0.26	2.30	1.27	0.02	0.15	0.04
DJ08	0.05	0.08	0.34	2.30	1.32	0.02	0.16	0.04
DJ09	0.05	0.07	0.38	2.37	1.34	0.02	0.18	0.04
DJ10	0.06	0.08	0.39	2.38	1.30	0.02	0.18	0.04
DJ11	0.05	0.07	0.39	2.38	1.39	0.02	0.18	0.04
DJ12	0.05	0.07	0.39	2.38	1.33	0.02	0.18	0.04
DJ13	0.05	0.07	0.40	2.40	1.33	0.02	0.18	0.05
DJ14	0.04	0.07	0.33	2.20	1.36	0.02	0.17	0.04
DJ15	0.04	0.07	0.32	2.17	1.39	0.02	0.16	0.05
DJ16	0.06	0.10	0.38	2.18	1.37	0.03	0.17	0.04
DJ17	0.05	0.08	0.39	2.19	1.41	0.02	0.17	0.05
DJ18	0.04	0.06	0.29	2.19	1.35	0.02	0.16	0.03
DJ19	0.07	0.07	0.27	2.19	1.34	0.02	0.18	0.05
DJ20	0.04	0.07	0.29	2.19	1.28	0.02	0.17	0.03
DJ21	0.04	0.07	0.29	2.11	1.33	0.02	0.16	0.03
DJ22	0.04	0.07	0.30	2.12	1.36	0.02	0.15	0.04
DJ23	0.04	0.07	0.29	2.20	1.36	0.02	0.15	0.04
DJ24	0.04	0.08	0.30	2.15	1.37	0.02	0.14	0.04

**sample ID**	**Cl^-**	**NO_3^-**	**SO_4^{2-}**	**HCO_3^-**	**Ca^{2+}**	**K^+**	**Mg^{2+}**	**Na^+**
				**mmol/l**				
DJ25	0.04	0.07	0.30	2.16	1.34	0.02	0.14	0.04
DJ26	0.03	0.08	0.28	2.17	1.32	0.02	0.15	0.04
DJ27	0.03	0.08	0.29	2.18	1.36	0.02	0.15	0.04
DJ28	0.03	0.08	0.30	2.20	1.38	0.02	0.15	0.04
DJ29	0.03	0.08	0.32	2.26	1.28	0.02	0.15	0.05
DJ30	0.04	0.09	0.36	2.35	1.33	0.02	0.16	0.04
DJ31	0.03	0.08	0.36	2.29	1.41	0.02	0.15	0.04
DH01	0.80	0.49	1.38	5.53	2.63	0.10	1.66	0.72
DH02	0.75	0.46	1.29	5.53	2.62	0.10	1.64	0.72
DH03	0.71	0.43	1.25	5.47	2.62	0.09	1.64	0.67
DH04	0.71	0.43	1.24	5.52	2.52	0.09	1.64	0.67
DH05	0.68	0.42	1.19	5.58	2.45	0.09	1.65	0.67
DH06	0.76	0.47	1.30	5.55	2.38	0.09	1.65	0.67
DH07	0.70	0.44	1.25	5.52	2.38	0.09	1.67	0.70
DH08	0.85	0.53	1.48	5.52	2.28	0.10	1.78	0.81
DH09	0.73	0.45	1.28	5.55	2.40	0.09	1.70	0.73
DH10	0.85	0.53	1.50	5.56	2.45	0.09	1.69	0.73
DH11	0.77	0.48	1.36	5.55	2.50	0.09	1.76	0.76
DH12	0.90	0.57	1.60	5.51	2.56	0.09	1.76	0.74
DH13	0.77	0.51	1.46	5.56	2.52	0.09	1.73	0.74
DH14	0.89	0.57	1.60	5.46	2.55	0.09	1.75	0.74
DH15	0.90	0.57	1.60	5.52	2.68	0.09	1.78	0.75
DH16	0.78	0.53	1.97	5.38	2.70	0.12	1.80	0.71
DH17	0.87	0.54	1.63	5.46	3.08	0.10	1.81	0.76
DH18	0.90	0.57	1.66	5.38	3.06	0.10	1.73	0.73

DH19	0.90	0.57	1.66	5.43	3.03	0.10	1.84	0.78
DH20	0.90	0.57	1.64	5.44	2.93	0.10	1.85	0.78
DH21	0.89	0.57	1.61	5.53	2.95	0.10	1.86	0.80
DH22	0.90	0.58	1.63	5.45	2.96	0.10	1.85	0.79
DH23	0.90	0.58	1.64	5.44	3.14	0.10	1.85	0.80
DH24	0.85	0.55	1.61	5.24	3.03	0.10	1.77	0.74
DH25	0.84	0.55	1.60	5.30	2.98	0.10	1.73	0.71
DH26	0.78	0.53	1.49	5.52	2.99	0.10	1.74	0.75
DH27	0.80	0.53	1.50	5.51	2.91	0.10	1.72	0.76
DH28	0.80	0.54	1.54	5.53	2.86	0.10	1.78	0.76
DH29	0.81	0.55	1.53	5.49	2.52	0.10	1.77	0.77
DH30	0.81	0.55	1.63	5.48	2.68	0.10	1.80	0.76
DH31	0.83	0.56	1.63	5.47	2.56	0.10	1.78	0.76
